# Possible permanent down regulation of oestrogen receptor expression during fetal life and breast cancer risk

**DOI:** 10.1038/sj.bjc.6690581

**Published:** 1999-07

**Authors:** J S Lawson, E Petridou, D Trichopoulos

**Affiliations:** Centre for Public Health, University of South Wales, Sydney, Australia; University of Athens, Medical School, Athens, Greece; Harvard School of Public Health, Boston, MA, USA


*British Journal of Cancer* (1999) **80**(10), 1678
© 1999 Cancer Research Campaign
Article no. bjoc.1999.0581

Letter to the Editor

### Possible permanent down regulation of oestrogen receptor expression during fetal life and breast cancer risk

### Sir

Oestrogen production rates in blood levels are higher among white American than among Chinese women (Bernstein et al, 1990) and higher oestrogen levels have been associated with higher risk for breast cancer (Lipworth et al, 1996; Toniolo, 1997). Thus, the recent finding (Lipworth et al, 1999) that oestrogens during pregnancy are higher among Chinese than among white American women appears to contradict the hypothesis that pregnancy oestrogens are positively associated with breast cancer risk in the offspring, because the incidence of breast cancer is five times higher among American than among Chinese women. A possible explanation, however, for the apparent contradiction is down-regulation of oestrogen receptor expression during fetal life in response to higher circulating levels of oestrogens. If this were to be true, one should expect lower expression of oestrogen receptors among Chinese women than among American women in response to higher circulating oestrogen levels in the former group.

Two lines of evidence suggest that this may, indeed, be the case. Oestrogen receptor expression in breast cancer tumours is much lower among Asian than among Caucasian breast cancer patients (Nomura et al, 1984; Stemmermann, 1991). Moreover, in a study of normal women in the UK, oestrogen receptor expression in normal breast tissue was substantially higher among European than among non-European women (Ricketts et al, 1991). Since exposure to sex steroids during perinatal life has irreversible effects in experimental animals (Verhoeven et al, 1982), the explanation for the profound differences in breast cancer incidence between Western and Oriental women may be the permanent down-regulation of oestrogen receptor expression in fetal human breast tissues, under the influence of variable levels of pregnancy oestrogens.

*JS Lawson*
*Centre for Public Health, University of South Wales, Sydney, Australia*

*E Petridou*
*University of Athens, Medical School, Athens, Greece*

*D Trichopoulos*
*Harvard School of Public Health, Boston, MA, USA*

### REFERENCES

Bernstein L, Yuan J-M, Ross RK, Pike MC, Hanisch R, Lobo R, Stanczyk F, Gao Y-T and Henderson BE (1990) Serum hormone levels in pre-menopausal Chinese women in Shanghai and white women in Los Angeles: results from two breast cancer case-control studies. *Cancer Causes Control* **1**: 51–58

Lipworth L, Adami H-O, Trichopoulos D, Carlstrom K and Mantoros CS (1996) Serum steroid hormone levels, sex hormone-binding globulin, and body mass index in the etiology of postmenopausal breast cancer. *Epidemiology* **7**: 96–100

Lipworth L, Hsieh C-C, Wide L, Ekbom A, Yu S-S et al (1999) Maternal pregnancy hormone levels in an area with high incidence (Boston) and low incidence (Shanghai, China) of breast cancer. *Br J Cancer* **79**: 7–12

Nomura Y, Tashiro H, Hamada Y and Shigematsu T (1984) Relationship between estrogen receptors and risk factors of breast cancer in Japanese pre and postmenopausal patients. *Breast Cancer Res Treat* **4**: 37–43

Ricketts D, Turnbull L, Ryall G, Bakshi R, Rawson NSB et al (1991) Estrogen and progesterone receptors in the normal female breast. *Cancer Res* **51**: 1817–1822

Stemmermann GN (1991) The pathology of breast cancer in Japanese women compared to other ethnic groups: a review. *Breast Cancer Res Treat* **18**: S67–S72

Toniolo PG (1997) Endogenous estrogens and breast cancer risk: the case for prospective cohort studies. *Environ Health Perspect* **105**: 587–592

Verhoeven, G, Vandoren G, Heyns W, Kuhn ER, Janssens JP et al (1982) Incidence, growth and oestradiol-receptor levels of 7,12-dimethylbenz(a)anthracene-induced mammary tumours in rats: effects of neonatal steroids and oestradiol implants. *J Endocrinol* **95**: 357–368

